# Prevalence of Unregistered, Substandard, and Falsified Veterinary Antimicrobials in Ethiopia: A National Postmarket Quality Surveillance Study

**DOI:** 10.1155/vmi/6524636

**Published:** 2026-07-29

**Authors:** Belachew Tefera, Belachew Bacha, Nardos Tefera, Gudeta Uma, Tadese Setegn, Bizuayehu Belete, Diriba Daba, Dinsefa Jemal, Yilak Tefera, Hussen Bedu, Tamiru Tilki, Dagnachew Hailemichael, Solomon Kebede, Hamid Jemal, Tesfa Marew, Dawit Teshome, Teferi Gedif, Esayas Gelaye, Tenaw Andualem, Ayenew Ashenef

**Affiliations:** ^1^ Animal Product and Input Quality Testing Center, Ethiopian Agricultural Authority, Akaki Kality District 07 P.O. Box 31303, Addis Ababa, Ethiopia; ^2^ Ethiopian Agricultural Authority, Addis Ababa, Ethiopia; ^3^ Department of Pharmaceutics and Industrial Pharmacy, School of Pharmacy, College of Health Sciences, Addis Ababa University, P.O. Box. 1176, Addis Ababa, Ethiopia, aau.edu.et; ^4^ Department of Social and Administrative Pharmacy, School of Pharmacy, College of Health Sciences, Addis Ababa University, P.O. Box. 1176, Addis Ababa, Ethiopia, aau.edu.et; ^5^ Food and Agricultural Organization of the United Nations, Ethiopia Country Office, P.O. Box: 5536, Addis Ababa, Ethiopia; ^6^ Department of Pharmaceutical Chemistry and Pharmacognosy, School of Pharmacy, College of Health Sciences, Addis Ababa University, P.O. Box. 1176, Addis Ababa, Ethiopia, aau.edu.et

**Keywords:** antimicrobial resistance, Ethiopia, postmarket quality surveillance, substandard and falsified (SF) veterinary medicines, veterinary pharmaceutical regulation

## Abstract

**Background:**

The proliferation of unregistered, substandard, and falsified (SF) veterinary pharmaceuticals represents a critical global threat to animal health, food security, and environmental conservation. In sub‐Saharan Africa, these products exacerbate the emergence of antimicrobial resistance (AMR), potentially accelerating the transition toward a “post‐antibiotic” era. Despite Ethiopia’s relatively developed regulatory framework compared to neighboring states, vulnerabilities such as inadequate diagnostic facilities, porous supply chains, and limited postmarketing surveillance results in enforcement persist.

**Objective:**

This descriptive study was conducted to determine the prevalence and quality profile of selected veterinary antimicrobials, including antibacterials, anthelmintics, and antiprotozoals, circulating in the Ethiopian market.

**Methods:**

Postmarket quality surveillance (PMS) study was conducted across five administrative regions. A total of 142 samples were collected and evaluated through three distinct regulatory tiers: verification of Ethiopian Agricultural Authority (EAA) registration status, standardized visual/physical screening, and official pharmacopeial (compendial) laboratory testing.

**Results:**

The majority of sampled products (95.7%) were imported, predominantly from China (75.7%) and India (15.0%). Primary sampling sites included veterinary retail outlets (50.7%) and clinics (22.9%). Regulatory analysis revealed that 17.9% (*n* = 25) of samples were unregistered. Visual screening identified an administrative substandard prevalence of 24.3% among registered products. Of the 44 samples subjected to compendial laboratory analysis, 22.7% (*n* = 10) failed to meet established pharmacopeial specifications. While suspected falsified products were identified through labeling discrepancies, laboratory confirmation of falsification is ongoing.

**Conclusions:**

Unregistered and substandard veterinary medicines are circulating in the Ethiopian supply chain, posing severe risks to animal health and contributing to the regional AMR crisis. These findings necessitate urgent, concerted efforts from stakeholders to strengthen the national regulatory system, enhance EAA laboratory capacity, and secure the pharmaceutical supply chain through targeted postmarketing enforcement.

## 1. Introduction

Ethiopia possesses the largest livestock population in Africa and the 10^th^ largest globally, with an estimated 70 million cattle, 43 million sheep, 52.5 million goats, 8.1 million camels, 13.3 million equines, and 57 million poultry [1]. Despite this vast biological capital, the sector’s economic contribution remains suboptimal due to systemic challenges, including traditional breeding practices, nutritional deficits, a fragmented animal healthcare infrastructure, and significant barriers to accessing quality‐assured, affordable veterinary medicinal products (VMPs) [2, 3].

For over six decades, VMPs have served a pivotal role in the prophylaxis, diagnosis, and treatment of animal diseases, as well as in growth promotion and productivity enhancement [4]. When utilized rationally—characterized by correct indication, appropriate dosage, and adherence to duration—VMPs yield substantial public health and socioeconomic benefits [5]. Conversely, irrational use, encompassing misuse, abuse, and the administration of **substandard and falsified (SF)** products, precipitates severe animal health crises, environmental degradation, and multifaceted economic losses [6]. Specifically, the circulation of poor‐quality antimicrobials is a primary driver of the global “post‐antibiotic” threat, accelerating the emergence and dissemination of antimicrobial resistance (AMR) [7].

The prevalence of SF veterinary medicines undermines international efforts in agricultural production, food security, and environmental conservation. A systematic review reported that approximately 6.5% of VMP samples from postmarket surveillance (PMS) studies failed at least one quality verification test [6]. Furthermore, a meta‐analysis of 20 surveys in low‐and‐middle‐income countries (LMICs) across Asia and Africa found that 52.0% of 1246 antimicrobial samples failed quality specifications, with out‐of‐specification active pharmaceutical ingredient (API) content, weight uniformity, and disintegration failure cited as the most prevalent defects. In fragmented settings, noncompliance rates for specific therapeutic classes have reached alarming levels: 11%–95% for antibiotics, 22%–58% for anthelmintics, and 28%–100% for trypanocides [8].

When SF veterinary drugs are used, of course not knowingly, it contributes to the rise of AMR, both undermining efforts to prevent, contain, or eradicate animal diseases, which are threats to animal and public health. The World Organization for Animal Health (WOAH) launched the substandard and falsified veterinary products (SFVPs) program in 2022. The program includes a pilot monitoring and surveillance initiative (VSAFE‐pilot) that tracks and reports unsafe veterinary products (TRUVET) in the system. These initiatives are targeted to counteract the problem of occurrences of SF veterinary medicines around the globe [9].

In Ethiopia, veterinary pharmaceuticals are regulated by the Ethiopian Agricultural Authority (EAA) under the Ministry of Agriculture, in accordance with Council of Ministers Regulation No. 509/2022 [10]. The regulatory functions include product registration and marketing authorization, licensing of manufacturers and importers, facility inspection, quality control (QC) and laboratory testing, PMS, and control of unauthorized products. PMS is a critical component, involving market inspections, sampling, laboratory testing, and pharmacovigilance. Marketing authorization holders are required to report adverse events and lack of efficacy; thus, regulatory actions such as recall or suspension may be taken when risks are identified [11]. PMS also includes monitoring antimicrobial use and residues in animal‐derived foods to address AMR, despite pharmacovigilance systems remaining underdeveloped in the country [12, 13]. Veterinary drug registration follows structured national guidelines, requiring preregistration compliance such as a certificate of competence and good manufacturing practice standards, submission of comprehensive dossiers covering quality, safety, and efficacy, and scientific evaluation by the authority. Approved products that have received a registration license are valid for five years, subject to renewal, while postapproval obligations include pharmacovigilance reporting and adherence to regulatory requirements [14]. Despite this comprehensive framework, implementation challenges persist in Ethiopia regarding veterinary medicine regulation, particularly in pharmacovigilance and regulatory enforcement, underscoring the need for system strengthening.

In Ethiopia’s veterinary healthcare system, antimicrobials are the drugs used to kill or inhibit the growth of microorganisms, including antibacterials, antifungals, antivirals, anthelmintics, and antiparasitics. Ethiopia has approved the second edition of the veterinary drugs list in 2019, which is arranged by pharmacotherapeutic classification that includes antimicrobials [15]. WOAH categorizes veterinary antimicrobials based on their importance to veterinary medicine and public health as veterinary critically important (VCIA), highly important (VHIA), and important (VIA) [16]. The most commonly used antimicrobials and their combinations as indicated on the list include **antibacterial** classes and their combinations: **tetracyclines** (e.g., oxytetracycline, doxycycline), **aminoglycosides** (e.g., dihydrostreptomycin, gentamycin); and **penicillins** (e.g., procaine penicillin G, cloxacillin + amoxicillin), and **sulfonamides** and their combinations (e.g., sulfadiazine, sulfdimidine, sulfamethoxazole–trimethoprim); **anthelmintics**: albendazole, ivermectin, and tetramisole; and **antiprotozoals**: diminazene aceturate, isomethamedium chloride, homidium, and diminazene diaceturate [17–19].

The 2017 data indicate that antimicrobial use in animals had accounted for approximately 73% of total global antimicrobial consumption. The widespread use of these agents for growth promotion and disease prevention in animals has significantly contributed to the development of AMR [20, 21]. Veterinary pharmaceuticals selected for this PMS study were identified based on several criteria, including high consumption and prescription rates, recurrent shortages and elevated prices, a documented history of quality concerns, and a potential risk of substandard and/or falsified products. These medicines are also among the top 10 most commonly used antimicrobials in the Ethiopian veterinary healthcare system [18, 19]. Furthermore, the volume of imported antimicrobials into the country substantially exceeds that of locally manufactured. The majority of these imports originate from Asia, particularly China and India, while a smaller proportion is sourced from Europe and the Middle East.

In Ethiopia, intensive antimicrobial use for growth promotion and long‐term prophylaxis is common, yet regulatory oversight is challenged by low public awareness, inadequate diagnostic facilities, and a porous supply chain [5]. Major systemic bottlenecks include inappropriate prescribing and dispensing practices, the proliferation of unregistered products, the presence of unlicensed retail outlets in rural regions, and the absence of a robust antibiotics utilization policy [1, 22]. Previous quality evaluations in Ethiopia have been limited by narrow geographical scopes or a focus on single product categories, hindering the extrapolation of national prevalence data. However, the escalating threat of SF products necessitates the implementation of risk‐based active and passive quality surveillance activities to inform strategic regulatory interventions.

While localized management issues, illicit cross‐border trade, and supply chain irregularities have been reported [23], the overall national prevalence of SF veterinary medicines in Ethiopia remains unestablished. This national survey was conducted by the EAA to establish a comprehensive quality profile and prevalence of SF products across three priority therapeutic categories: antibacterials, anthelmintics, and antiprotozoal agents.

## 2. Materials and Methods

### 2.1. Study Design and Regulatory Framework

A descriptive cross‐sectional study was conducted to evaluate the quality and registration status of VMPs in Ethiopia. The study followed the PMS guidelines established by the EAA, which was harmonized with the World Health Organization (WHO) guidelines for conducting surveys on medicine quality.

The investigation was executed in three phases: **Phase-I (preparation)** protocol development, validation of data collection instruments, and recruitment/training of data collectors, **Phase-II (implementation)** field‐based sample procurement and collection, and **Phase-III (analysis)** visual and physical screening, registration verification, and compendial laboratory testing.

A technical working group comprising experts from the EAA, the Food and Agriculture Organization (FAO), and academic institutions provided oversight on study scope, sampling strategy, and logistical coordination.

### 2.2. Sampling Strategy and Procurement

#### 2.2.1. Selection of Sampling Sites

**FIGURE 1:**
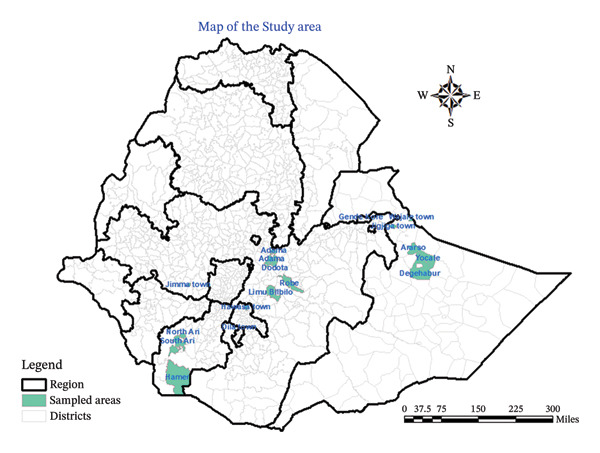
A map of Ethiopia depicts the study areas where veterinary antimicrobial samples were collected from the market. The map was created with QGIS software Version 3.38.0.

A multistage sampling technique was employed to ensure geographic and agro‐ecological representation. Regional states were stratified into three production systems: urban agriculture (Dire Dawa), mixed crop–livestock (Oromia, SNNPR, and Sidama), and pastoralist systems (Somali), as shown in Figure 1.

Lot quality assurance sampling (LQAS) was applied using a binomial framework with predefined thresholds of acceptable quality (*p*_1_ = 5%) and unacceptable quality (*p*_2_ = 20%). A sample size of 19 units per lot was employed, with a decision rule of d = 0, whereby the detection of at least one substandard or falsified product resulted in the classification of the lot as unacceptable. Five regions were included in the study; accordingly, the minimum required sample size was 95 (19 samples per lot × 5 lots), consistent with WHO guidelines for medicine quality surveys. Although a minimum sample size of 100 is generally considered methodologically robust for such studies, a total of 142 samples were collected to enhance the reliability and representativeness of the findings [24].

Regions were selected using a combination of purposive sampling (based on livestock population and market volume) and random sampling. Accordingly, the Oromia, Sidama, and Somali Regions were included alongside SNNPR. Regional capitals and one zone with the highest livestock density per region were targeted. Within these zones, districts (Woredas) were selected based on ecological stratification: *Kola* (arid lowlands), *Woyna Dega* (temperate), and *Dega* (cold highlands). Convenience sampling was used to choose final sampling sites, which included wholesalers, clinics, pharmacies, and farms (community and commercial).

#### 2.2.2. Targeted Veterinary Pharmaceuticals

VMPs were selected based on high consumption volume, risk of shortage, price volatility, and historical quality concerns. The study focused on three priority therapeutic categories: (1) **antibacterials:** oxytetracycline 20% injection, penicillin–streptomycin 20/20, 20/25 injections, and co‐trimoxazole 200/40 mg injection; (2) **anthelmintics:** albendazole 2500 mg bolus, tetramisole 600 mg bolus, and ivermectin 1% injections; and (3) **antiprotozoals:** diminazene diaceturate + phenazone (444 + 556 mg) powder.

### 2.3. Quality Evaluation and Laboratory Analysis

#### 2.3.1. Registration Verification and Visual Screening

Quality evaluation was conducted in a three‐stage hierarchical process. Initially, product registration experts verified the samples against the EAA national database. Abstracted data included registration status, API, country of origin, and shelf‐life metadata. Visual screening was conducted using a standardized WHO checklist to assess **(a) packaging integrity:** layout, container security, and secondary packaging; **(b) labeling compliance:** presence of registration numbers, manufacturer details, and designated local agents; and **(c) falsification indicators:** any labeling discrepancies or physical defects suggestive of falsified origin. Samples failing initial administrative screening were categorized as “unregistered” or “substandard by packaging” and were generally excluded from further laboratory testing unless required for specific forensic analysis.

#### 2.3.2. Compendial Confirmatory Testing

Due to the resource‐intensive nature of pharmacopeial analysis, a risk‐based approach was utilized for laboratory evaluation. In‐depth physical inspection (morphology, color, and dimensions) was conducted by QC experts. Following preliminary identification, a prioritized subset of samples was subjected to compendial confirmatory testing at the EAA Quality Control Laboratory. Analysis included **API content**/**assay, pH determination, sterility, and microbial limit tests (MLTs)** using official pharmacopeial (USP/BP/IP) or validated in‐house methods. Results were classified as “compliant,” “substandard,” or “falsified” based on their adherence to established specifications.

### 2.4. Statistical Analysis

All collected data were cleaned, coded, and entered into the **Statistical Package for the Social Sciences (SPSS) Version 20.0** (IBM Corp., Armonk, NY, USA). Descriptive statistics, including frequencies and percentages, were utilized to summarize product characteristics, geographic distribution, registration status, and quality failure rates. Where applicable, cross‐tabulations were performed to evaluate the relationship between product origin (local vs. imported) and regulatory compliance.

### 2.5. Operational Definitions

The study adopted the following standardized definitions for VMPs, harmonized with the WHO and contemporary regulatory literature:

• **Substandard products:** Also referred to as “out‐of‐specification” (OOS) products, these are authorized veterinary medicines that have undergone the formal EAA registration process but fail to meet established quality standards or pharmacopeial specifications due to manufacturing errors, degradation, or poor storage practices.
• **Unregistered/unlicensed products:** VMPs that have not been evaluated or approved for marketing, distribution, or clinical use by the national regulatory authority. These products circumvent legal importation channels and lack valid marketing authorization.
• **Falsified products:** Medical products that involve a deliberate and fraudulent misrepresentation of their identity, composition, or source. This includes products with the correct ingredients but fraudulent packaging, those with incorrect APIs, or those with no API at all.

## 3. Results

### 3.1. General Characteristics of Collected Samples

A total of 142 veterinary medicine samples were initially procured for the study based on the accessibility of sampling sites and drug availability. After excluding two samples due to incomplete data abstraction, 140 samples were included in the final analysis. The geographical and pharmaceutical distribution of these samples is summarized in Table 1.

**TABLE 1:** Distribution of veterinary medicine samples by source, supply chain level, geographical region, and dosage form (*N* = 140).

Variable		Frequency (*n*)	Percentage (%)
Product source	Imported	134	95.7
	Locally manufactured	4	2.9
	Unidentified	2	1.4

Supply chain level	Veterinary drug retail outlet	71	50.7
	Veterinary clinic	32	22.9
	Informal outlets	18	12.9
	Wholesaler	17	12.1
	Other^a^	2	1.4

Geographic region	Somali	47	33.6
	SNNPR^b^	41	29.3
	Oromia	36	25.7
	Sidama	13	9.3
	Dire Dawa	3	2.1

Dosage form	Solution for injection	72	51.4
	Bolus	37	26.4
	Granules^∗^	16	11.4
	Suspension for injection	14	10.0
	Others^∗^	1	0.7

^a^Samples from regional veterinary laboratories or distribution hubs.

^b^South Nations, Nationalities, and Peoples’ Region.

^∗^Granules and others refer to diminazene diaceturate + phenazone samples, which are presented as granules to be dissolved for injection and others as complete solutions for injection. In Table 2, this antiprotozoal medicine is put in a separate line.

#### 3.1.1. Geographical Distribution and Sampling Sites

The samples were collected across five administrative regions, with the highest concentration obtained from the Somali Region (33.6%, *n* = 47), followed by the SNNPR (29.3%, *n* = 41). Regarding the point of purchase within the supply chain, private veterinary drug retail outlets served as the primary source, accounting for 50.7% (*n* = 71) of the samples, followed by veterinary clinics (22.9%, *n* = 32).

#### 3.1.2. Product Origin and Pharmaceutical Form

The veterinary medicine market in the study areas was found to be heavily reliant on international trade; the majority of samples were imported (95.7%, *n* = 134) with only a marginal proportion representing local manufacturing. In terms of formulation, solutions for injection were the most prevalent dosage form, representing over half of the collected samples (51.4%, *n* = 72), followed by boluses (26.4%, *n* = 37). Dire Dawa is an urban center where limited husbandry and veterinary practice exist, and its geographical area is also very limited compared to other sampling collection sites; thus, only three samples were collected.

**TABLE 2:** Distribution of collected veterinary medicine samples by therapeutic category and brand diversity (*N* = 140).

Therapeutic category	Active ingredient and dosage form	*n* (%)	No. of brands
Anthelmintics		71 (50.7)	44
	Ivermectin 1% (injection)	31 (22.1)	21
	Albendazole 2500 mg (bolus)	22 (15.7)	9
	Tetramisole 600 mg (bolus)	18 (12.9)	14

Antimicrobials		52 (37.1)	28
	Oxytetracycline 20% (injection)	26 (18.6)	15
	Penicillin–streptomycin (injection)^a^	20 (14.3)	12
	Cotrimoxazole (injection)^b^	6 (4.2)	1

Antiprotozoals		17 (12.1)	13
	Diminazene diaceturate + phenazone^c^	17 (12.1)	13

Total		**140 (100.0)**	**85**

*Note: n* = frequency. Bold values indicate that 140 shows the total samples, 100 shows the percent, and 85 shows the total number of samples in terms of brands.

^a^Procaine penicillin and dihydrostreptomycin (20/20 or 20/25).

^b^Sulfamethoxazole + trimethoprim (200/40 mg).

^c^444 + 556 mg powder for injection.

### 3.2. Therapeutic Categories and Common Active Ingredients

Analysis of the collected drugs by therapeutic class revealed that anthelmintics constituted the highest proportion of the collection, accounting for 50.7% (*n* = 71) of the total samples. This was followed by antimicrobials at 37.1% (*n* = 52) and antiprotozoals at 12.1% (*n* = 17). As detailed in Table 2, the most frequently encountered APIs across these categories were ivermectin (anthelmintic), albendazole (anthelmintic), and oxytetracycline (antimicrobial). The high prevalence of these specific medicines shows their critical role in the management of endemic parasitic and bacterial diseases in Ethiopia’s livestock sector.

### 3.3. Product Origin and Registration Status

The VMPs sampled in this study were predominantly sourced from international manufacturers, with China (75.7%, *n* = 106) and India (15.0%, *n* = 21) serving as the primary countries of origin. Regulatory evaluation against the EAA database revealed that while the majority of samples (80.7%, *n* = 113) were formally registered, a notable proportion (17.9%, *n* = 25) were circulating without valid marketing authorization. Data abstraction was incomplete for two samples (1.4%. *n* = 2).

A critical vulnerability in the supply chain was identified regarding imported products: 80.0% (*n* = 20) of all unregistered medications originated from China. Detailed cross‐tabulations of registration status by country of origin are provided in Table 3.

**TABLE 3:** Cross‐tabulation of veterinary medicine samples by country of origin and regulatory registration status (*N* = 139).

Country of origin	Registered (*n*)	Unregistered (*n*)	Total (*N*)	Proportion (%)
China	86	20	106	76.8
India	20	1	21	15.2
Ethiopia (local)	4	0	4	2.9
Greece	0	2	2	1.4
Netherlands	2	0	2	1.4
Belgium	1	0	1	0.7
Korea	1	0	1	0.7
Unidentified	2	0	2	1.4
Total	**116**	**23**	**139**	**100.0**

*Note:* One sample was excluded from the original 140 due to missing data. Bold values indicate the total in each subcategory.

### 3.4. Distribution of Unregistered Products by Therapeutic Class

Among the medications circulating without valid EAA marketing authorization (*n* = 25), specific formulations within the anthelmintic and antimicrobial categories were identified as high‐risk. As illustrated in Table 4, the most frequently encountered unregistered veterinary medicines were ivermectin (1% injection), which accounted for 32.0% (*n* = 8) of all unregistered samples; oxytetracycline (20% injection), which represented 28.0% (*n* = 7) of the unregistered cohort; and tetramisole (600 mg bolus), which comprised 20.0% (*n* = 5) of the unauthorized products. Collectively, these three products represented 80.0% (*n* = 20) of the total unregistered samples identified in the survey, highlighting a significant concentrated gap in regulatory oversight for high‐demand injectable and bolus formulations.

**TABLE 4:** Distribution of unregistered veterinary medicine samples by active ingredient and dosage form (*n* = 25).

Active pharmaceutical ingredient (API)	Dosage form	*n* (%)
Ivermectin (1%)	Solution for injection	8 (32.0)
Oxytetracycline (20%)	Solution for injection	7 (28.0)
Tetramisole (600 mg)	Bolus	5 (20.0)
Penicillin–streptomycin^a^	Suspension for injection	3 (12.0)
Albendazole (2500 mg)	Bolus	1 (4.0)
Diminazene diaceturate + phenazone^b^	Powder for injection	1 (4.0)
Total		**25 (100.0)**

*Note:* *n* = frequency of unregistered samples.

^a^Combination of procaine penicillin and dihydrostreptomycin (20/20 or 20/25 mg/mL).

^b^444 mg diminazene diaceturate + 556 mg phenazone.

### 3.5. Visual Screening and Regulatory Compliance

The visual inspection and administrative review conducted by EAA experts identified significant deviations from national regulatory standards. Of the 140 samples analyzed, 17.9% (*n* = 25) were confirmed to be unregistered, circulating without valid marketing authorization.

#### 3.5.1. Administrative and Labeling Noncompliance

Among the samples that were officially registered, the prevalence of products classified as substandard due to labeling or administrative deficiencies was 24.3% (*n* = 28) of the total 115 samples. As illustrated in Table 5, several critical omissions were identified as the following:

**TABLE 5:** Administrative and labeling noncompliances identified during visual screening (*N* = 115).

Category of noncompliance	Parameter of deviation	Frequency (*n*)	Percentage (%)
Registration and agency	Absence of registration number on package	64	55.7
	Absence of local agent information	49	42.6
	Registered products without local agent	24	20.9
	Different local agent than the registration database	2	1.7

Packaging and labeling	No package insert provided	13	11.3
	Absence of manufacturer name/information	8	7.0
	No secondary packaging	5	4.3
	No batch number	5	4.3
	Different package inserts than the database	5	4.3
	Package insert not intact	2	1.7
	Absence of pack size	1	0.9

Product specifications	Different shelf‐life description	6	5.2
	Different strength than the database	4	3.5
	Different dosage form description	4	3.5
	Different active ingredient (API) description	4	3.5
	Different manufacturer than the database	4	3.5
	Different brand/trade name than the database	4	3.5
	Different generic name description	3	2.6
	Different dose/kg instructions	2	1.7
	Different route of administration	1	0.9

Physical integrity	Product color non‐uniformity	6	5.2
	Different storage condition requirements	2	1.7
	Different physical appearance than the database	2	1.7

*Note: N* = 115 refers to registered samples with evaluable data. Percentages may exceed 100%, as a single sample could exhibit multiple noncompliances.

Primary deficiencies: The most prevalent violations were the absence of a national registration number (55.7%, *n* = 64) and the omission of a designated local agent (42.6%, *n* = 49) on the outer packaging.

Minor noncompliances: Less frequent deviations included discrepancies in the stated route of administration compared to the EAA database and the absence of specified pack sizes, each occurring in 0.9% (*n* = 1) of the noncompliant cases.

These findings suggest that even within the legal supply chain, a significant portion of veterinary medicines fail to meet the mandatory labeling requirements set forth by the EAA, potentially complicating pharmacovigilance and product traceability (see Table 6).

**TABLE 6:** Summary of regulatory compliance (N = 140).

Regulatory indicator	*n*/*N*	Rate (%)
Unregistered samples (total population)	25/140	17.9
Overall suspected substandard (administrative)	53/140	37.9
Substandard registered samples	28/115	24.3

### 3.6. Technical Visual Inspection and QC Assessment

Comprehensive visual screening was conducted by quality assurance and control (QA/QC) experts to evaluate the physical and administrative integrity of the samples against established national criteria of requirements for veterinary medicines.

#### 3.6.1. Parameters of Full Compliance

The assessment revealed high levels of adherence to essential product specifications. All sampled units **(100%)** demonstrated full compliance with the following criteria:

• Physical integrity: Uniformity of color, absence of surface defects (cracks or cap irregularities), and absence of observable particulate contaminants.
• Essential labeling: Correct description of generic names (APIs), dosage strengths, and clearly legible expiration dates.

#### 3.6.2. Identified Technical Noncompliances

Despite the high physical integrity of the products, specific regulatory and technical omissions were prevalent. The most significant noncompliances included:

• Analytical specifications: Of the 120 samples, 84.5% (*n* = 120) did not mention the official pharmacopeial methods for analysis (e.g., BP, USP, or IP).
• Regulatory representation: 35.9% of regulatory representation (*n* = 51) did not include mandatory local agent information on primary or secondary packaging.
• Supplemental information: 12.2% (*n* = 17) of the samples were distributed without the mandatory package inserts that offer critical pharmacological information about the medicine.

#### 3.6.3. Overall QC Failure Rate

Of the 142 total samples subjected to in‐depth visual inspection, 25.4% (*n* = 36) failed to meet the aggregate established QC criteria. Detailed results of the specific deviations per sample are provided in Table 7, with the full checklist available in the Supporting File.

**TABLE 7:** Results of in‐depth visual screening of veterinary medicine samples by quality control experts (*N* = 142).

Category	Visual inspection criteria	Compliant (*n*, %)	Noncompliant (*n*, %)
Physical integrity	Uniformity of color	142 (100.0)	0 (0.0)
	Absence of surface defects	142 (100.0)	0 (0.0)
	Absence of physical damage	140 (98.6)	2 (1.4)

Labeling and information	Generic name (API) indicated	142 (100.0)	0 (0.0)
	Strength mentioned on label	142 (100.0)	0 (0.0)
	Expiration date indicated	142 (100.0)	0 (0.0)
	Storage conditions mentioned	141 (99.3)	1 (0.7)
	Manufacturing date indicated	141 (99.3)	1 (0.7)
	Dosage form type indicated	140 (98.6)	2 (1.4)
	Pack size mentioned	139 (97.9)	3 (2.1)
	Brand/trade name described	136 (95.8)	6 (4.2)
	Similarity between primary and secondary labels	128 (90.1)	14 (9.9)

Administrative and regulatory	Manufacturer information complete	132 (93.0)	10 (7.0)
	Secondary packaging present	131 (92.3)	11 (7.7)
	Package insert available	124 (87.3)	18 (12.7)
	Local agent information indicated	91 (64.1)	51 (35.9)
	Official analysis method described (e.g., USP, BP)	22 (15.5)	120 (84.5)

*Note: n* = number of samples.

### 3.7. Compendial Laboratory Analysis Results

A subset of 44 samples underwent rigorous laboratory evaluation to assess compliance with official pharmacopeial specifications. Testing parameters included active ingredient assay, pH determination, sterility, and MLTs. The analytical methods employed included high‐performance liquid chromatography (HPLC) for the determination of ivermectin, albendazole, and oxytetracycline, following United States Pharmacopeia (USP) methods with appropriate analytical method verification and instrument qualification. Titrimetric analysis was used for penicillin G, while UV–visible spectrophotometry was applied for tetramisole. In addition, microbiological assay methods were utilized for the analysis of dihydrostreptomycin. All instruments used in the analysis were calibrated and qualified, and analytical procedures were conducted in compliance with established quality assurance protocols to ensure reliability and reproducibility of results. The laboratory had obtained ISO 17025 accreditation for testing of veterinary pharmaceuticals recently.

Typical representative HPLC chromatograms obtained in the experiments in this study had been shown in Figure 2.

FIGURE 2Typical HPLC chromatograms for ivermectin, albendazole, and oxytetracycline. (A) Ivermectin sample chromatograms: (I) Reference standard, (II) Sample 02, (III) Sample 01, and (IV) Sample 03. (B) Albendazole chromatograms: (I) Sample 02, (II) Sample 03, (III) Sample 01, and (IV) reference standard. (C) Oxytetracycline: (I) Sample 01, (II) reference standard (III) Sample 02.

**Figure:**
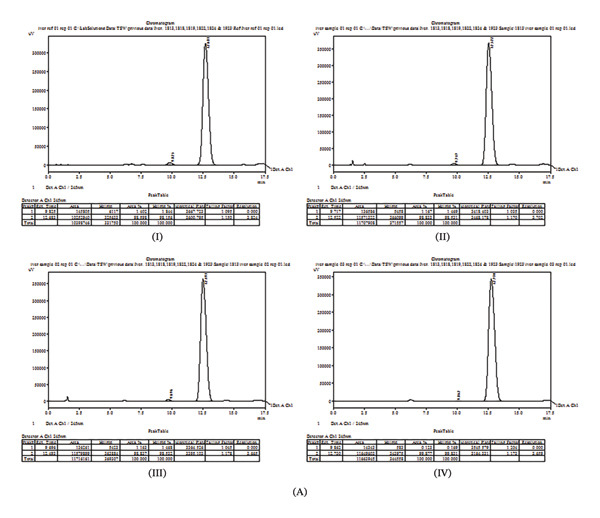

**Figure:**
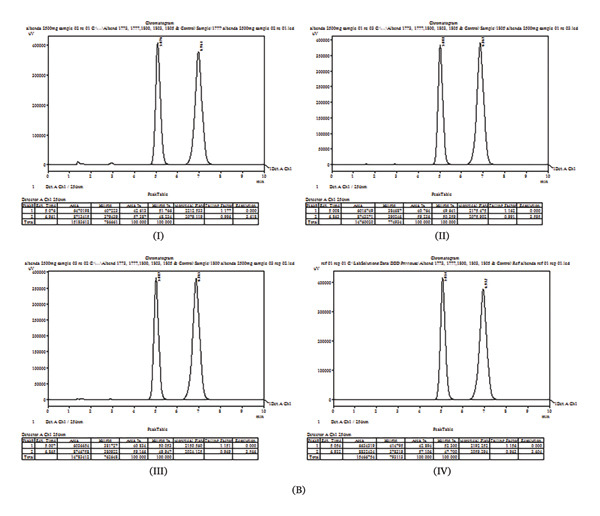

**Figure:**
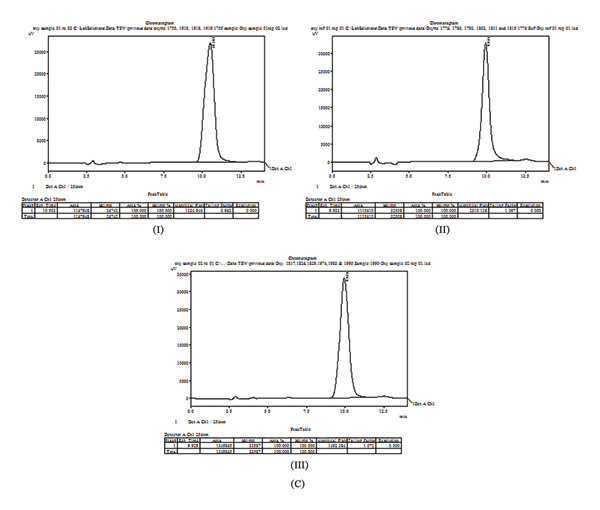

#### 3.7.1. Laboratory Failure Rates and Defect Classification

As detailed in Table 8, 22.7% (*n* = 10 of the total 44 samples) of the tested samples failed to meet established regulatory specifications. The primary drivers of laboratory failure were as follows: assay noncompliance 80.0% (*n* = 8) of the failures were attributed to subpotent or super‐potent active ingredient levels; and microbiological failure 20.0% (*n* = 2) of the samples failed the MLT, indicating contamination or inadequate preservative efficacy.

**TABLE 8:** Distribution of laboratory test failures by product type and specific compendial parameter (*n* = 10).

Product description	Frequency (*n*)	Assay failure (*n*)	MLT failure (*n*)
Ivermectin (1% injection)	6	6	0
Albendazole (2500 mg bolus)	3	1	2
Penicillin–streptomycin (20/20 injection)^a^	1	1	0
Total	**10**	**8**	**2**

*Note: n* = number of substandard samples. Bold values indicate the total in each subcategory.

^a^Procaine penicillin and dihydrostreptomycin.

#### 3.7.2. Product‐Specific Substandard Prevalence

The distribution of substandard findings was concentrated within specific therapeutic agents: ivermectin (1% injection), whose formulation exhibited the highest failure rate, accounting for 60.0% (*n* = 6) of all laboratory‐confirmed substandard samples. Albendazole (2500 mg bolus) represented 30.0% (*n* = 3) of the noncompliant products. Penicillin–streptomycin (20/20 injection) showed the lowest failure rate, with only 10.0% (*n* = 1) found to be substandard.

The high failure rates observed for ivermectin align with previous reports indicating the drug’s widespread anticipation for COVID‐19 treatment and symptom management [25, 26]. This surging demand created supply chain gaps, incentivizing manufacturers to produce substandard formulations that often contained insufficient levels of the API [27, 28]. Additionally, these therapeutic failures may stem from inherent storage and stability vulnerabilities characteristic of the drug [28, 29].

Generally, the root cause for poor‐quality veterinary pharmaceuticals’ existence in the supply chain might arise due to one or a combination of the following: (I) a manufacturing error that includes unit operations like weighing, mixing, and other steps; (II) nonconformity to CGMP standards; (III) degradability over time; and (IV) incorrect formulation. The errors might be intentional or non‐intentional.

#### 3.7.3. Geographic Distribution of Confirmed Substandard Samples

The regional distribution of samples that failed laboratory testing revealed significant geographic variance: **SNNPR (** *n* = 5), **Oromia** (*n* = 3), **Somali (** *n* = 1), and **Dire Dawa** (*n* = 1). These findings emphasize the need for targeted regulatory interventions in regions with higher concentrations of substandard products and reinforce the importance of routine EAA laboratory surveillance to ensure the quality of high‐demand veterinary medications like ivermectin.

## 4. Discussion

Access to quality‐assured VMPs remains a critical challenge in resource‐constrained regions. In sub‐Saharan Africa (SSA), the supply chain is heavily dependent on imports, as local manufacturing remains nascent. While countries such as Ethiopia, Nigeria, Sudan, Angola, and Kenya have established limited production of conventional dosage forms and vaccines, the vast majority of the region relies on international markets [30]. This restricted access has historically facilitated the proliferation and spread of SF products and the expansion of illegal pharmaceutical trade, with estimates suggesting that 20%–30% of veterinary medicines in SSA circulate through illicit channels [31]. In 2017 HealthforAnimals, the Global Animal Medicines Association, estimated that the annual worldwide veterinary products trade was worth US$30 billion. However, nearly 3% of approved veterinary products were either falsified, substandard, unregistered/unauthorized, illegally compounded products, illegal vaccines, or intellectual property rights counterfeits [32].

### 4.1. Regulatory Status and Market Authorization

In the present study, 17.9% (*n* = 25) of the collected samples were found to be unregistered with the EAA. While significant, this prevalence is notably lower than that of Nigeria, where 70% of animal owners reported using unregistered medicines due to price and availability constraints [33], and Kenya, where inadequate access encourages the use of nonlicensed products [34]. Despite the lower relative prevalence in Ethiopia, the circulation of unauthorized medicines poses severe risks, such as treatment failure, the introduction of contaminants into the human food chain, and broader threats to biodiversity and public health.

### 4.2. Visual Screening and Administrative Compliance

Visual inspection by regulatory experts revealed that **24.3%** of registered samples were substandard. The most frequent deficiencies were the absence of a national registration number (**55.7%**) and the omission of a local agent on the label (**42.6%**). These findings contrast favorably with mainland Tanzania, where 92% of samples failed product information requirements [35], and West African studies in Mali (43%) and Cameroon (68%) [36].

Interestingly, previous data from the Ethiopian Animal Products and Inputs Quality Testing Center (API‐QTC) laboratory reported a lower labeling defect rate of 1.3% [1]. This discrepancy is likely due to the nature of the samples: API‐QTC data often involve preregistration samples submitted by manufacturers, whereas this study utilized postmarket samples collected directly from the supply chain, which are more representative of the actual quality available to customers.

### 4.3. Compendial Analysis and Therapeutic Implications

Pharmacopeial laboratory testing revealed that **22.7%** of tested samples did not meet at least one quality specification (assay, pH, or microbial limit). **Ivermectin (1% injection)** emerged as the most problematic product, representing **60%** of all laboratory failures. These results are consistent with localized studies in Gondar, Ethiopia, which reported high failure rates (up to 80.95%) for anthelmintics [37], and Addis Ababa, where 30% of albendazole brands failed assay tests [38].

As a result of these assay failures, livestock are exposed to subtherapeutic concentrations of antimicrobials and anthelmintics, which is a major cause and driver of AMR spread and emergence. This phenomenon not only compromises animal health and productivity but also threatens national food security and degrades public trust in the veterinary healthcare system [8].

The SF veterinary medicine problem plausibly leads to adverse health impacts for animals and animal health, farming communities, and beyond. Globally, data on SF veterinary medicines are scattered, complicating efforts to understand their epidemiology and impact at the global level. Based on minimal study findings, WOAH in 2020 reported that 11%–95% of antibiotics (based on nine studies), 22%–58% of anthelmintics (4 studies), and 28%–100% of trypanocides (7 studies) used in the veterinary health sector are SF [39]. Another meta‐analysis conducted by Vidhamaly et al. as per 20 included studies found that 52% of the 1246 veterinary medicine samples collected in Asia and Africa that were tested for quality were SF. According to this systematic review and meta‐analysis report, 46.6% of the sample’s active ingredient content was out‐of‐specification, and 4.2% contained incorrect active ingredient(s); these were the most common reasons for sample failures [38]. Whereas, in the Ethiopian study, 1.3% of tested products showed defects in physical characteristics, packaging, or labeling information, while 6.9% of samples of the investigated products failed to comply with the pharmacopoeias’ and supplier’s specification limits set for assay. Of those that failed the pharmacopeias and suppliers’ specifications, a significantly higher sample number (90.1%) did not comply with the minimum assay specification limit [1]. A recent unpublished systematic review and meta‐analyses that incorporated 13 published works, which included 4 studies from Ethiopia, estimated that the overall prevalence of SF veterinary drugs in Africa stands at 37.54% [40]. The SF prevalence results found in this study are comparatively less than this value. However, as the goal is to make it near zero and avail safe and quality veterinary pharmaceuticals, it should not be interpreted as a success but rather as a springboard to perform extra efforts to circumvent the challenge.

### 4.4. Study Limitations

Several factors limit the generalizability of these findings. Due to logistical and financial constraints, the survey could not encompass all regions and city administrations. Furthermore, the capacity of the EAA laboratory restricted the scope of compendial testing. Critical parameters such as dissolution, disintegration, content uniformity, impurity profiles, and endotoxin levels were not assessed. The absence of these rigorous tests suggests that the true prevalence of substandard products in the market might be higher than reported here.

## 5. Conclusion and Recommendations

This national survey underscores significant challenges regarding the quality and regulatory compliance of veterinary pharmaceuticals in the Ethiopian market. Visual and physical evaluations revealed a prevalence rate of **25.4% (36/142)** for suspected SF products. These findings were further corroborated by compendial laboratory analysis, where **22.7% (10/44)** of tested samples failed to meet official pharmacopeial specifications.

While current premarket testing of lots and consignments remains a foundational practice, these results demonstrate that such measures are insufficient to secure the supply chain. To mitigate the risks associated with SF veterinary medicines and based on the study results, the following strategic interventions are recommended:

• **Digitalization of the supply chain**: The EAA should fully implement the digital VMP registration system to enable end‐to‐end tracking of veterinary medicines. The newly launched online registration platform (January 2025) should be integrated with customs and regional livestock bureaus to ensure that only high‐quality medications enter the market.
• **Strengthened PMS:** The EAA should transition from reactive testing to regular, risk‐based, and targeted postmarket quality surveillance. This would not only improve data collection but also serve as a deterrent against the distribution of illicit medicinal products.
• **Technological integration:** The EAA should prioritize the deployment of rapid screening technologies (such as TLC‐based Minilabs) at major ports of entry and regional hubs. This would allow for the immediate detection of suspect products and reduce the burden on official compendial testing laboratories.
• **Enforcement of labeling standards:** Stricter penalties must be imposed on importers and distributors whose products fail to display mandatory information, specifically clear EAA registration numbers and designated local agent contact information on primary and secondary packaging.
• **Deterrence measures:** The EAA should develop and enforce clear, punitive measures for noncompliance to serve as an effective deterrent against the circulation of unauthorized or mislabeled veterinary medicines.
• **Quality management systems (QMS)**: Establishing a comprehensive QMS within the EAA will provide a standardized approach to identifying, reporting, and removing SF products from the market, ultimately safeguarding animal health and national food security.

By implementing these strategic measures, Ethiopia can better protect its livestock sector, ensure food safety, and mitigate the public health risks associated with AMR driven by poor‐quality veterinary medicines. In conclusion, addressing the gaps identified in this survey requires a multifaceted approach that combines improved technical capacity, digitalized data management, and proactive field enforcement to ensure that only safe and effective veterinary medicines reach the end‐users.

**Nomenclature**
AMR: Antimicrobial resistance
API: Active pharmaceutical ingredient
APIQTC: Animal Products and Input Quality Testing Center
BP: British Pharmacopoeia
EAA: Ethiopian Agricultural Authority
FAO: Food and Agriculture Organization of the United Nations
IP: International Pharmacopoeia
ISO/IEC: International Organization for Standardization / International Electrotechnical Commission
LIMS: Laboratory information management system
LMICs: Low‐ and middle‐income countries
MLT: Microbial limit test
OOS: Out‐of‐specification
PMS: Postmarket surveillance
QA/QC: Quality assurance/quality control
QMS: Quality management system
SF: Substandard and falsified
SNNPR: South Nations, Nationalities, and Peoples′ Region
SPSS: Statistical Package for the Social Sciences
SSA: Sub‐Saharan Africa
TLC: Thin‐layer chromatography
USP: United States Pharmacopeia
VMP: Veterinary medicinal product
WHO: World Health Organization
WOAH: World Organization for Animal Health (formerly OIE)

## Funding

This study was financially supported by the Food and Agriculture Organization of the United Nations (FAO) and the Ethiopian Agricultural Authority (EAA) under the framework of the National Veterinary Drug Quality Surveillance Program.

## Disclosure

While the funding agencies provided technical oversight and logistical coordination during the study design and data collection phases, they had no direct role in independent laboratory analysis tasks, data interpretation, or the final decision to submit the manuscript for publication.

## Ethics Statement

The study protocol was reviewed and formally approved by the Technical Working Group of the Ethiopian Agricultural Authority (EAA). Because the research focused exclusively on the quality and registration status of commercially available VMPs and did not involve direct experimentation on live animals or human subjects, formal institutional animal ethics or biosafety committee approval was deemed not applicable. All procurement activities were conducted in accordance with the national PMS guidelines of Ethiopia. Verbal informed consent was obtained from all facility owners or authorized representatives at the points of purchase prior to sample collection.

## Conflicts of Interest

The authors declare no conflicts of interest.

## Supporting Information

Additional supporting information can be found online in the Supporting Information section.

## Supporting information


**Supporting Information** Data abstraction tool for the study.

## Data Availability

The datasets generated and analyzed during the current study are included within this manuscript. However, specific identifying details regarding proprietary brand names, individual manufacturers, and sampled facilities have been anonymized or withheld to maintain regulatory and commercial confidentiality. Requests for further technical data may be directed to the corresponding author or the Ethiopian Agricultural Authority (EAA) subject to institutional approval.
